# Comparative study on the wound healing effects of local versus systemic application of Polymyxin B in mice infected with multidrug-resistant *Pseudomonas aeruginosa*

**DOI:** 10.3389/fmicb.2026.1812925

**Published:** 2026-05-18

**Authors:** Chenhu Ma, Dan Wang, Bingjie Zhang, Jianshe Xu, Miaomiao Lin, Yishan Zheng, Yuxin Guo

**Affiliations:** 1 Department of Intensive Care Unit, The Second Hospital of Nanjing, Affiliated to Nanjing University of Chinese Medicine, Nanjing, China; 2 School of Public Health, Nanjing Medical University, Nanjing, China; 3 Department of Intensive Care Unit, Peking University Shenzhen Hospital, Shenzhen, China

**Keywords:** extensively drug-resistant, mouse model, Polymyxin B, *Pseudomonas aeruginosa*, wound infection

## Abstract

**Background:**

Wound infections caused by extensively drug-resistant *Pseudomonas aeruginosa* (XDR-PA) pose a significant clinical challenge with extremely limited treatment options. Polymyxin B (PMB) represents one of the last therapeutic lines of defence, yet its optimal route of administration (topical, intravenous, or combined) for deep tissue infections such as wounds remains to be further investigated.

**Objective:**

This study aimed to investigate the efficacy differences of different PMB administration routes (topical, intravenous, and combined therapy) in treating XDR-PA wound infections in mice, providing experimental evidence for optimizing clinical treatment regimens.

**Methods:**

One hundred and twenty Kunming mice were randomly assigned to five groups (*n* = 24 per group): (1) sham-operated group (uninfected); (2) infected untreated group; (3) infected + topical administration group (5 mg/kg); (4) Infection + intravenous administration group (5 mg/kg); (5) Infection + combined topical and intravenous administration group (2. 5 mg/ kg each). A full-thickness dorsal skin defect wound infection model was established in mice. Drug administration was in initiated 24 h post- infection and was performed once daily for four consecutive days. Daily observations and recordings were made of mouse body weight changes and wound erythema. Every 4 days post-infection, three mice were randomly selected and euthanized from each group for wound tissue collection to determine bacterial load. On days 6 and 17, wound tissue was collected for protein content analysis, cytokine (e.g., IL-1β, TNF-*α*) detection, and histopathological assessment.

**Results:**

Compared to the untreated infected group, all administered treatment groups demonstrated therapeutic efficacy. The topical administration group exhibited the most pronounced wound healing promotion, it achieved the highest wound closure rate, fastest resolution of erythema and oedema, and optimal bacterial clearance efficiency. Additionally, it showed effective regulation of inflammatory response, and minimal histopathological damage. The intravenous administration group exhibited the next highest efficacy. The combination therapy group did not demonstrate synergistic effects superior to local monotherapy. Mice in the untreated infection group exhibited the slowest weight gain and delayed wound healing. Mice in the untreated infection group exhibited the slowest weight loss and delayed wound healing.

**Discussion (Conclusion):**

Under the experimental conditions employed, topical application of PMB demonstrated superior efficacy to intravenous administration and combined therapy in treating wound infections in XDR-PA mice. Due to the equal total daily dose design (5 mg/kg for all treatment groups), the combination therapy group received a lower dose per route (2.5 mg/kg each) compared to the monotherapy groups (5 mg/kg via a single route). Therefore, no definitive conclusion can be drawn regarding potential synergistic or antagonistic effects of combination therapy based on this study design. This suggests that targeted local administration may represent a more effective and cost-efficient strategy for localized infections caused by resistant bacteria, which warrants further clinical translational research.

## 1 Introduction

*Pseudomonas aeruginosa* is an opportunistic Gram-negative pathogen renowned for its intrinsic and acquired antimicrobial resistance. It is a primary causative agent of hospital - acquired infections, particularly wound infections in burn, trauma, and immunocompromised patients (Horcajada et al., 2019; Pachori et al., 2019). This bacterium forms biofilms, which are bacterial colonies embedded within an extracellular polymeric matrix, a structure that confers significant resistance to antimicrobial agents and host immune responses, thereby leading to chronic and difficult-to-treat infections (Costerton et al., 1999; Ciofu and Tolker-Nielsen, 2019).

The emergence and spread of extensively drug-resistant *Pseudomonas aeruginosa* (XDR-PA) represent a severe public health crisis globally. This crisis has been driven by the misuse of broad-spectrum antibiotics such as carbapenems (Bassetti et al., 2018). Extensively drug-resistant (XDR) strains typically resist all conventional antibiotics except polymyxins, leaving clinical treatment options severely limited (Livermore, 2002; López Causapé et al., 2018). Against this backdrop, polymyxin B(PMB), a cationic polypeptide antibiotic derived from *Baci lus polymyxinogenes,* has re-emerged as the ‘last line of defence’ against MDR Gram-negative bacterial infections due to its ability to disrupt the outer membrane of these pathogens (Nation et al., 2016; Tsuji et al., 2019).

The clinical application of PMB faces two major challenges: firstly, its inherent nephrotoxicity and neurotoxicity, which limit systemic dosing and treatment duration (Kelesidis and Falagas, 2015; Eljaaly et al., 2021); secondly, its significantly reduced efficacy when treating biofilm-associated infections. At standard doses, PMB penetrates biofilms poorly and may fail to eliminate dormant and persistent bacteria. This often leads to treatment failure and recurrent infection (Jørgensen et al., 2024; Pamp et al., 2008). To overcome these limitations, researchers are exploring localized delivery strategies for PMB. Topical administration (e.g., local irrigation, wound dressings) delivers high drug concentrations directly to the infection site while minimizing systemic exposure. This approach potentially reducing toxicity and enhancing efficacy (Ozhathil and Wolf, 2022). Preclinical studies have demonstrated promising outcomes with topical PMB in treating pulmonary and burn infection models (Lin et al., 2017).

Nevertheless, direct, systematic comparative studies evaluating different PMB administration routes, particularly systemic versus topical delivery, in treating pan-resistant *Pseudomonas aeruginosa* wound infections remain scarce. While conventional systemic administration (e. g., intraperitoneal or intravenous injection) may control systemic sepsis, it may fail to achieve effective bactericidal concentrations within the local wound environment (Lin et al., 2018). Conversely, while topical administration achieves high local concentrations, its efficacy in controlling deep tissue infections and potential systemic spread remains uncertain (Bouchene et al., 2018). Furthermore, the potential impacts of both routes on wound healing processes, local inflammatory responses, and bacterial biofilm clearance remain poorly elucidated.

Against this backdrop, systematically evaluating the relative efficacy of different PMB administration routes in a pan-resistant *Pseudomonas aeruginosa* wound infection model has critical translational value. Determining whether local administration is superior, equivalent, or serves as an adjunct to systemic therapy will provide key experimental evidence for optimizing clinical treatment regimens.

This study uses a mouse full-thickness skin defect wound infection model to simulate clinically relevant multidrug-resistant *Pseudomonas aeruginosa* infections. It will systematically compare the therapeutic outcomes of PMB administered via systemic routes (e.g., intravenous injection), topical routes (e.g., direct wound application), and combined systemic and topical administration. We comprehensively evaluate the antimicrobial efficacy and safety of different administration strategies by assessing a series of indicators, including bacterial load, changes in mouse body weight, histopathological alterations, inflammatory cytokine levels, and wound healing rates.

We hypothesize that topical application of PMB or combined systemic and topical administration will more effectively eradicate multidrug-resistant *Pseudomonas aeruginosa* and its biofilm within wounds, reduce local tissue damage, and promote wound healing compared to systemic administration alone, while demonstrating superior safety. The findings of this study may offer clinicians novel and more effective therapeutic strategies and theoretical support for managing challenging wound infections caused by multidrug-resistant *Pseudomonas aeruginosa*.

## 2 Materials and methods

### 2.1 Bacterial strain, antibiotic and experimental animals

A clinical, XDR-PA strain PA36 (GenBank number: PRJNA1424245) was used in this study. The strain, stored in our laboratory repository, was confirmed to be resistant to most tested antimicrobial categories except polymyxins, as determined by the broth microdilution method in accordance with Clinical and Laboratory Standards Institute (CLSI) guidelines (Belley et al., 2025). The minimum inhibitory concentration (MIC) of PMB sulfate (Shengong Bioengineering Co., Ltd., Shanghai, China) against this strain was 2 μg/ mL. For intravenous administration, a PMB stock solution was prepared fresh in sterile physiological saline.

A total of 120 specific pathogen-free (SPF), 7-week-old Kunming mice, with an initial body weight of 32 ± 2 g, were used in this 30-day study. Kunming mice, an outbred strain widely used in China, were selected for this study due to their cost-effectiveness, robust fertility, and well-documented resistance to opportunistic infections, which facilitates the establishment of a chronic wound infection model without premature systemic sepsis. However, we acknowledge that the use of outbred mice limits direct comparison with studies predominantly employing inbred strains (e.g., C57BL/6, BALB/c).Mice were purchased from Henan Skebes Biotechnology Co., Ltd. (Henan, China) and housed in standard cages within a clean, pathogen-free animal facility. They were provided with irradiated standard rodent diet and distilled water ad libitum. Environmental conditions were maintained at 24–26 ° C, 30–60% relative humidity, under a 12-h light/dark cycle. All animal procedures were performed in strict accordance with the National Research Council’s Guide for the Care and Use of Laboratory Animals (8th edition) and followed the ARRIVE guidelines 2.0 for reporting animal research. The experimental protocol was approved by the Animal Ethics Committee of Kangtai Medical Laboratory Services Hebei Co., Ltd. (Approval No. MDL 2025-04-17-01). All surgeries were performed under sodium pentobarbital anesthesia, and every effort was made to minimize animal suffering. All animal procedures were performed in compliance with the National Research Council’s Guide for the Care and Use of Laboratory Animals and were approved by the Animal Ethics Committee of Kangtai Medical Laboratory Services Hebei Co., Ltd. (Approval No. MDL 2025-04-17-01).

### 2.2 Mouse model of extensively drug-resistant *Pseudomonas aeruginosa* wound infection

A murine full-thickness excisional wound infection model was established as previously described, with minor modifications (Watters et al., 2013). After a one-week acclimatization period, 120 mice were randomly allocated into five experimental groups (*n* = 24 per group): (1) Uninfected control (Control); (2) Infected, untreated control (INF); (3) Infected, topical PMB treatment [PMB(TOP)]; (4) Infected, intravenous PMB treatment [PMB(IV)]; (5) Infected, combined topical and intravenous PMB treatment [PMB(TOP+IV)]. Mice were anaesthetized by intraperitoneal injection of sodium pentobarbital (50 mg/kg). The dorsal hair was removed using electric clippers and a depilatory cream, and the skin was disinfected with iodophor. A full-thickness, circular excisional wound (8 mm in diameter) was created using a sterile biopsy punch, and the wound edges were trimmed with sterile surgical scissors to standardize the wound bed.

For infection, a 50 μL suspension of the XDR-PA strain PA36 (4 × 108 CFU/mL in logarithmic growth phase) was applied directly to the wound bed. The wound was then covered with a sterile semi-occlusive dressing (3 M ™ Tegaderm™) for 24 h to facilitate initial bacterial colonization. Successful wound infection was confirmed using the following predefined criteria: (i) bacterial load ≥ 10^6^ CFU/g tissue on day 7 post-infection; (ii) mean wound healing rate ≤30% in the untreated infected (INF) group on day 6; (iii) local infection severity score (erythema, edema, exudate, crust; each 0–3, total 0–12) ≥ 6 sustained for at least 5 consecutive days in the INF group; and (iv) survival rate ≥90% in the INF group on day 10. All infected groups met these criteria, confirming successful infection establishment. Failure of infection (spontaneous clearance) was not observed in any infected mouse. Mice in the uninfected Control group received an equal volume of sterile saline instead of the bacterial inoculum. Postoperative analgesia was provided via a sugar-containing jelly for pain relief, energy supplementation, and palatability enhancement. Ample feed and water access was maintained (Gates et al., 2023).

### 2.3 Antibiotic treatment protocol

Dissolve PMB sulfate in sterile saline solution. Treatment should commence 24 h after infection. Using a micropipette, apply 50 μL of the solution evenly to the surface of the wound, then gently spread it with sterile gauze to ensure the solution covers the entire wound. After allowing the solution to air-dry naturally for approximately 2 min, a new layer of sterile semi-occlusive dressing (3 M Tegaderm) is applied to maintain wound moisture and prevent the m ice from licking the wound. Treatment is administered once daily for 4 consecutive days. This topical administration method mimics the clinical practice of wound irrigation or instillation therapy. The dosing regimen was designed as follows: PMB (TOP) and PMB (IV) monotherapy groups: received a dose of 5 mg/kg/day via their respective routes. PMB (TOP+IV) combination therapy group received a total daily dose of 5 mg/kg, equally split between the two routes (2 0.5 mg/kg via topical application and 2.5 mg/kg via intravenous injection). This design is intended for preliminary exploration only and cannot serve as a basis for evaluating synergistic effects. Rationale for the isodose design: This approach aimed to compare the efficacy of administration routes independent of the total drug load. It specifically to test for synergistic interactions rather than effects from increased cumulative dosing, while simultaneously modeling a potential clinical strategy to reduce systemic drug exposure and associated toxicity (Tsuji et al., 2019). Mice in the uninfected Control and infected, untreated INF groups received equivalent volumes of sterile saline via both topical application and intravenous injection as placebo controls.

### 2.4 Monitoring of body weight and wound healing

Body weight and wound dimensions were monitored to assess systemic health and local healing progression. Starting 24 h post-infection (designated as Day 0), the body weight of each mouse was recorded every 2 days. At the same intervals, wounds were photographed alongside a calibrated scale, and the wound length and width were measured using a digital caliper. The wound area was calculated from these measurements using ImageJ software (Version 1.8.0, National Institutes of Health, United States; Schneider et al., 2012). The wound healing rate for each group at each time point was calculated using the following formula: Wound Healing Rate (%) = [(Ao __ An) / Ao] × 100% where Ao represents the initial wound area on Day 0, and An represents the residual wound area on post-operative day *n*.

### 2.5 Quantification of bacterial burden in wound tissue

To quantify the bacterial load, wound tissue samples were collected for microbiologica analysis at regular intervals. On postoperative days 4, 8, 12, and 16, three mice were randomly selected and euthanized from each group. A full-thickness section of the wound (approximately 0.5 g) was aseptically excised, weighed, and placed in a sterile tube containing 4.5 mL of sterile 1 × phosphate-buffered saline (PBS). The tissue was homogenized thoroughly using an electric tissue homogenizer for 5–10 min to create a 1:9 (w/v) tissue homogenate. This standardized homogenization protocol ensures uniform bacterial release from the tissue matrix for accurate colony counting (Olson et al., 2002). Subsequent serial dilutions and plating were performed as per standard microbiological procedures. For each wound tissue homogenate, three technical replicates were plated, and the mean colony-forming unit (CFU) count was calculated. Thus, each data point represents three biological replicates (*n* = 3 mice per group per time point) with three technical replicates per sample.

### 2.6 Histological processing of wound tissue

For histopathological analysis, wound tissue samples were collected at designated endpoints. Following euthanasia by sodium pentobarbital overdose (150 mg/kg), a full-thickness section of the wound and surrounding skin was aseptically excised. Tissue samples were immediately fixed in 4% paraformaldehyde (PFA) solution (Biosharp, Beijing, China) for 24–48 h at 4 ° C. Fixed tissues were then dehydrated through a graded series of ethanol and cleared in xylene. They were subsequently and embedded in paraffin wax following a standard protocol (Fischer et al., 2008). Serial sections of 3 μm thickness were cut using a rotary microtome, mounted on glass slides, and dried overnight at 60° C prior to staining.

### 2.7 Histopathological and special staining

For general histopathological assessment, deparaffinized sections were stained with hematoxylin and eosin (H& E) following a standard protocol (Fischer et al., 2008). Briefly, sections were deparaffinized in xylene, rehydrated through a graded ethanol series, stained with hematoxylin, differentiated, blued, and counterstained with eosin. After dehydration and clearing, sections were mounted with a resinous medium. Stained sections were examined and imaged using an upright light microscope (Olympus BX53, Olympus Corporation, Japan).

Collagen deposition and tissue morphology were assessed using Masson’s trichrome staining (Suvik and Effendy, 2012). Deparaffinized sections were stained successively with Weigert’s iron hematoxylin, Bieb rich scarlet-acid fuchsin, phosphomolybdic-phosphotungstic acid solution, and aniline blue. Following the manufacturer’s instructions (Masson Trichrome Stain Kit, Solarbio, China). This stains collagen fibers blue, muscle fibers and cytoplasm red, and nuclei dark blue. After dehydration and mounting, sections were examined under a light microscope.

To visualize bacterial morphology and distribution within tissues, Giemsa staining was performed (Difford, 2002). Deparaffinized sections were stained with Giemsa working solution for 5 min, differentiated in phosphate buffer (pH 6. 8), and rinsed gently with distilled water. After air-drying, sections were mounted and examined under a light microscope using an oil immersion lens (100 × objective).

### 2.8 Immunohistochemical analysis

Immunohistochemistry was performed to localize the expression of interleukin-1 *β* (IL-1 β), tumor necrosis factor-alpha (TNF-*α*), and transforming growth factor-beta (TGF-β) in wound tissues. Deparaffin ized and rehydrated sections underwent antigen retrieval by heating in citrate buffer (pH 6. 0) for 30 min in a microwave oven. Endogenous peroxidase activity was blocked with 3% hydrogen peroxide for 10 min at room temperature. After washing with phosphate-buffered saline (PBS), sections were incubated with a protein block (normal goat serum). This step was performed for 30 min to reduce non-specific binding.

Sections were then incubated overnight at 4 ° C with primary antibodies against mouse IL-1β (Cell Signaling Technology, #12242, 1:200), TNF-α (Abcam, ab6671, 1:250), or TGF-β (Santa Cruz Biotechnology, sc-146, 1: 100). According to the A lz Forum antibody database, this antibody is a rabbit polyclonal antibody immunized with the C-terminal peptide of human TGFβ1; the sequence of this peptide is identical to that of mouse TGFβ1, and the antibody has been validated for cross-reactivity in humans, mice, rats and pigs (Alz Forum, 2024).

Our laboratory used mouse spleen tissue as a positive control to confirm the specificity of this antibody in mouse tissue. All staining batches included both positive and negative controls (omitting the primary antibody). Following PBS washes, sections were incubated with a biotinylated secondary antibody (Vector Laboratories) for 1 h at room temperature. They were then incubated with streptavidin-horseradish peroxidase (HRP) complex (Vector Laboratories) for 30 min. Positive signals were developed using 3,3’-diaminobenzidine(DAB) as the ch romogen (DAB Substrate Kit, Vector Laboratories), and the reaction was stopped by immersion in distilled water. Nuclei were counterstained with hematoxylin. Finally, sections were dehydrated, cleared in xylene, and mounted with a permanent mounting medium.

Appropriate positive and negative (omission of primary antibody) controls were included in each staining run to ensure specificity. All steps were performed following established I HC protocols (Ramos Vara, 2005; Magaki et al., 2019). Stained sections were examined and imaged under a light microscope (Olympus BX53).

### 2.9 Enzyme- linked immunosorbent assay

Concentrations of total protein (TP), hydroxyproline (HYP), and cytokines (IL-1*β*, TNF-*α*, TGF-β) in wound tissues were quantified using enzyme-linked immunosorbent assay (ELISA).

Tissue samples collected on postoperative days 6 and 17 were homogenized in cold PBS containing protease inhibitors. The homogenates were centrifuged at 3,000 × *g* for 10 min at 4 ° C. The supernatants were collected and stored at −80 ° C until analysis.

The concentrations of the target analytes were measured using commercial, mouse-specific ELISA kits according to the manufacturers’ instructions, as follows: Total protein (TP): Protein Content Assay Kit (BCA method, 48 samples, spectrophotometry; ADS-F-SP002, ADS Biotechnology Co., Ltd., Shanghai, China); Hydroxyproline (HYP): Hydroxyproline Assay Kit (acid hydrolysis method, 48 samples, spectrophotometry; ADS-F-AJS008, ADS Biotechnology Co., Ltd., Shanghai, China); IL-1β: Mouse Interleukin-1β ELISA Kit (48 T; MK2776B, Meilian Biotechnology Co., Ltd., Shanghai, China); TNF-α: Mouse Tumor Necrosis Factor Alpha ELISA Kit (48 T; MK2868B, Meilian Biotechnology Co., Ltd., Shanghai, China); TGF-β1: Mouse Transforming Growth Factor Beta 1 ELISA Kit (48 T; MK2871B, Meilian Biotechnology Co., Ltd., Shanghai, China).” For each assay, the optical density (OD) at 450 nm was measured using a microplate reader. Cytokine concentrations (pg/ mg) were determined by interpolation from a standard curve. They were normalized to the total protein content (mg/ mL) of each sample to account for variations in tissue cellularity, as is standard practice in tissue homogenate analysis (Banks, 2000). Each tissue sample was measured in triplicate technical replicates, and the average value was used. For each group at each time point, three mice (*n* = 3 biological replicates) were analyzed.

### 2.10 Statistical analysis

All data are expressed as mean ± standard deviation (SD). Each group at each time point comprised three biological replicates (mice), with each biological replicate undergoing three technical replicates, the results of which were averaged. First, the Shapiro–Wilk test was used to assess normality for each dataset, and Levene’s test was used to assess homogeneity of variances. All data were normally distributed and exhibited homogeneity of variances (*p* > 0.05). For comparisons between multiple groups, one-way ANOVA was employed; for data involving two factors (e.g., body weight changes, wound healing rates), two-way repeated measures ANOVA was used. When ANOVA indicated significant differences, Tukey’s HSD (honestly significant difference) *post-hoc* test was performed for multiple comparisons. All statistical analyses were performed using Graph Pad Prism 10.1.2 software. The significance level was set at *p* < 0.05. Significance symbols in the figures: ****p ≤ 0.0001, ***p ≤ 0.001, **p ≤ 0.01, *p ≤ 0.05, ns: *p* > 0.05.

## 3 Results

### 3.1 Differential effects of PMB administration routes on wound healing and systemic health in XDR-PA infected mice

**Figure 1:**
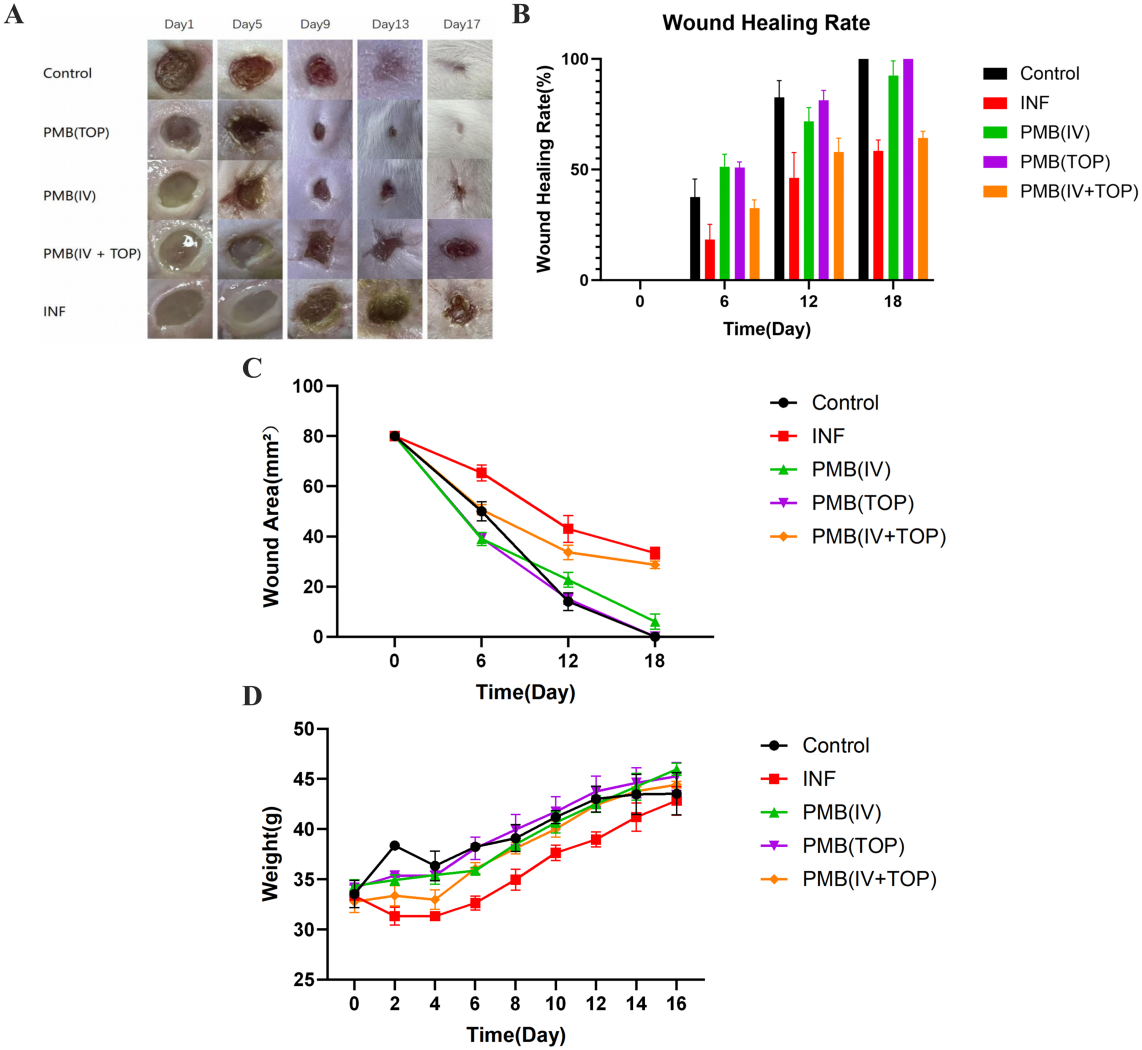
Differential effects of Polymyxin B (PMB) administration routes on wound healing and systemic health in XDR-PA infected mice. **(A)** Representative photographs of the wounds in each group of mice, taken on days 1, 5, 9, 13 and 17 following infection. **(B)** Wound healing rates in each. **(C)** Wound healing area in each group of mice following surgery. **(D)** Weight changes in each group of mice every 2 days following surgery. Data are presented as mean ± SD; *n* = 3 biological replicates (mice) per group per time point, each with three technical replicates.

Wound healing progression and systemic health were monitored following infection with extensively drug-resistant *Pseudomonas aeruginosa* (XDR-PA) PA36 and subsequent treatment. Serial wound photography (Figure 1A) documented visual healing outcomes. By day 5 post-infection, wound contraction and scab formation were initiated in the non-infected Control, topical PMB [PMB(TOP)], and intravenous PMB [PMB(IV)] groups. A pronounced reduction in wound size was evident in the PMB (TOP) group by days 9 and 13, with the Control and PMB (IV) groups also showing progressive closure. By day 17, wounds in the Control and PMB (TOP) groups were completely healed, with full re-epithelialization and hair regrowth, while wounds in the PMB(IV) group were nearly resolved. In contrast, wound healing in the combined topical and intravenous group [PMB(TOP+IV)] was significantly delayed. The infected, untreated control group (INF) showed persistent clinical signs of active infection throughout the observation period, including suppuration, erythema, and edema.

Quantitative planimetric analysis (Figures 1B,C) corroborated the photographic observations. On day 6, wound healing was initiated in all groups. The PMB (IV) and PMB (TOP) monotherapy groups showed comparable and superior initial healing rates (51% each). These rates were significantly higher than those of the Control (37.5%), PMB (TOP+IV; 32.5%), and INF (18%) groups. From days 12 to 18, the PMB (TOP) group maintained the most rapid healing trajectory, closely matching the Control group, followed by the PMB (IV) group. Although the absolute healing rates of the INF and PMB (TOP+IV) groups increased over time, their progression remained significantly slower. By the study endpoint (day 18), the Control and PMB (TOP) groups achieved complete wound closure (100%). The PMB (IV), PMB (TOP+IV), and INF groups reached closure rates of 92.5, 64.2, and 58.3%, respectively. Final wound area measurements (day 18, Figure 1C) confirmed this hierarchy, with residual areas of 0 mm2 (Control and PMB(TOP)), 9 mm2 (PMB(IV)), 28.7 mm2 [PMB(TOP+IV)], and 33.3 mm2 (INF).

Body weight, a key indicator of systemic well-being, was monitored throughout the experiment (Figure 1D). While all groups exhibited an overall trend of weight gain, the rate of increase differed significantly. Mice in the INF and PMB (TOP+ IV) groups gained weight at a markedly slower pace compared to the Control, PMB (TOP), and PMB (IV) groups, with the most pronounced deceleration observed in the INF group. This indicates that both persistent XDR-PA infection and the specific dosing regimen employed in the combination therapy group exerted a measurable negative impact on the systemic physiological status of the animals.

### 3.2 Bacterial burden in wounds is differentially reduced by PMB administration routes

**Figure 2:**
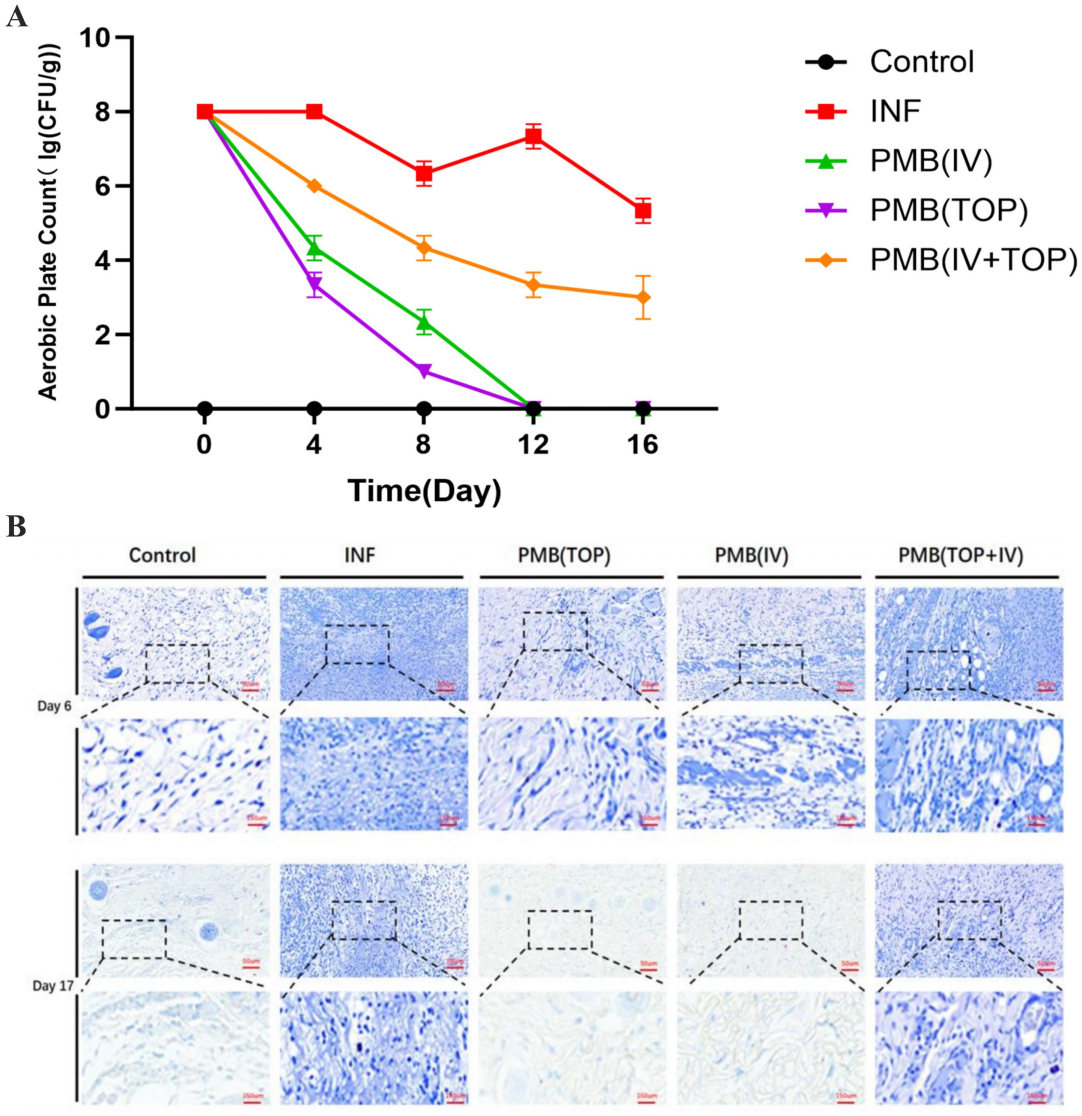
The effect of different routes of administration on bacterial load in wounds. **(A)** Colony counts of *Pseudomonas aeruginosa* (PA36) in the postoperative wounds of mice in each group. **(B)** Giemsa staining of wound tissue in mice on days 6 and 17 post-surgery.

The bacterial load at the wound site was dynamically influenced by the route of PMB administration (Figure 2A). As expected, wounds in the uninfected Control group remained sterile throughout the 16-day observation period. In contrast, the infected, untreated group (INF) sustained persistently high bacterial counts. Colony-forming units (CFU) declined to approximately 6.3 log10 CFU/g by day 8, then rebounded to approximately 7.3 log10 CFU/g by day 12. This pattern confirmed the establishment of a resilient, chronic infection model characteristic of XDR-PA.

Monotherapy with PMB, via either intravenous [PMB(IV)] or topical [PMB(TOP)] routes, led to a progressive reduction in bacterial counts from day 4 onwards. The PMB(IV) group demonstrated a rapid initial decline, achieving an approximate 4.0 log10 CFU/g reduction compared to the INF group by day 8. The PMB (TOP) group exhibited the most potent and rapid bactericidal effect. It achieved a reduction of nearly 5.3 log10 CFU/g versus the INF group by day 8, with counts falling to 1.0 log10 CFU/g. This antimicrobial effect was sustained. Bacterial loads in both monotherapy groups approached the detection limit by day 12, indicating near-sterilization of the wound bed. Statistical analysis confirmed that bacterial burdens in the PMB(IV) and PMB(TOP) groups were significantly lower than in the combined therapy group [PMB(TOP+IV)] from day 4 onward (*p* < 0.0001). They were also consistently lower than in the INF group at all post - treatment time points (*p* < 0.0001). These results underscore the superior and sustained bactericidal activity of the monotherapy regimens, particularly topical administration. This route achieved high local drug concentrations and persistent bacterial clearance.

Histological assessment via Giemsa staining (Figure 2B) corroborated the quantitative culture data. At days 6 and 17, abundant bacteria were visibly present within the wound tissues of the INF and PMB (TOP+IV) groups. In contrast, bacterial presence was markedly diminished or absent in the PMB (TOP) and PMB (IV) groups at corresponding time points. No bacteria were detected in the Control group.

In summary, *P. aeruginosa* established a persistent infection in untreated wounds. This contributed to chronic inflammation. Both topical and intravenous PMB monotherapy were highly effective in suppressing and eradicating the pathogen. In this model, local administration demonstrated the most significant therapeutic effect.

### 3.3 Regulatory effects of PMB on total protein content and granulation tissue formation in wound tissue

**Figure 3:**
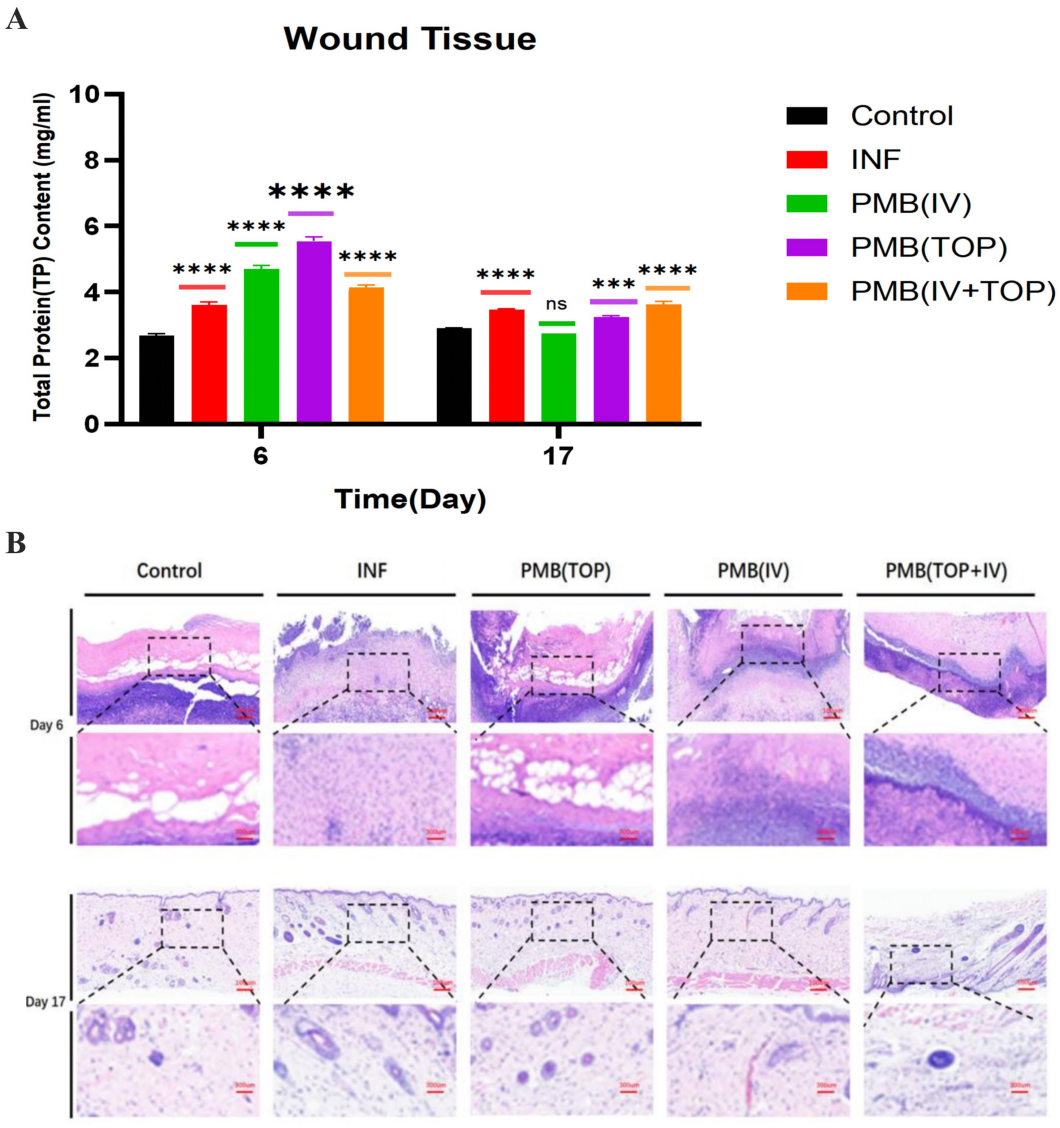
The effect of PMB on total protein content in wound tissue and granulation tissue formation. **(A)** Total protein (TP) content in wound tissue from mice in each group on days 6 and 17 post-surgery. Data are presented as mean ± SD; *n* = 3 biological replicates (mice) per group per time point, each with three technical replicates (****p ≤ 0.0001, ***p ≤ 0.001, **p ≤ 0.01, *p ≤ 0.05, ns: *p* > 0.5). **(B)** Hematoxylin and eosin staining was performed on wound tissue from the mice on days 6 and 17 post-surgery.

During wound healing, the total protein (TP) concentration in wound tissue homogenates, which serves as a comprehensive indicator of cell density and exudative response, was significantly regulated by infection and different treatment regimens (Figure 3A). In the early phase (day 6), XDR-PA infection in the INF group induced a marked increase in TP (3.6 mg/ mL) compared to the uninfected control group (2 0.7 mg/ mL). This reflects the protein - rich exudate and cellular infiltration characteristic of acute infection. All PMB-treated groups exhibited further elevated TP levels at this stage, with the highest concentrations observed in the PMB (TOP) group (5.5 mg/mL) and PMB (IV) group (4.7 mg/ mL). This indicated a persistent inflammatory response superimposed upon early reparative cellular activity. By the late stage (day 17), a marked shift was observed. The INF group exhibited persistently elevated TP levels (3 0.5 mg/ mL), indicating ongoing inflammation and delayed progression to the late healing phase. In contrast, all treatment groups showed reduced TP values compared to day 6. The most pronounced decreases were observed in the PMB (IV) and PMB (TOP) groups, with TP levels falling to 2.8 and 3.2 mg/mL respectively, values matching or falling below those of the INF group. The decrease in total protein levels (particularly in monotherapy groups) coincided with the resolution of acute inflammation and oedema. This reflected the transition to the tissue remodeling phase and indicating effective infection control.

Histological assessment of granulation tissue via H&E staining revealed a structural correlation with the total protein count data obtained from wound tissue homogenates (Figure 3B). On day 6, the INF group exhibited dense neutrophilic infiltration, marked oedema, and necrosis, with only sparse, disorganized granulation tissue visible at wound margins. Compared to the control and PMB (TOP) groups, the PMB (IV) and PMB (IV + TOP) groups showed a sparser dermal matrix. They also had less developed granulation tissue at this early time point. By day 17, increased granulation tissue deposition was observed in all groups. However, granulation tissue in the control, PMB (IV), and PMB (TOP) groups was thicker and showed higher cellular density than in the INF and PMB(IV + TOP) groups. The combined data indicate that persistent *Pseudomonas aeruginosa* infection disrupts normal protein dynamics. It also impedes granulation tissue maturation. During the initial treatment phase, topical monotherapy with PMB [PMB(TOP)] creates a t issue environment more conducive to early repair. This is superior to intravenous administration or combination therapy.

### 3.4 PMB administration modulates collagen deposition and tissue remodeling

**Figure 4:**
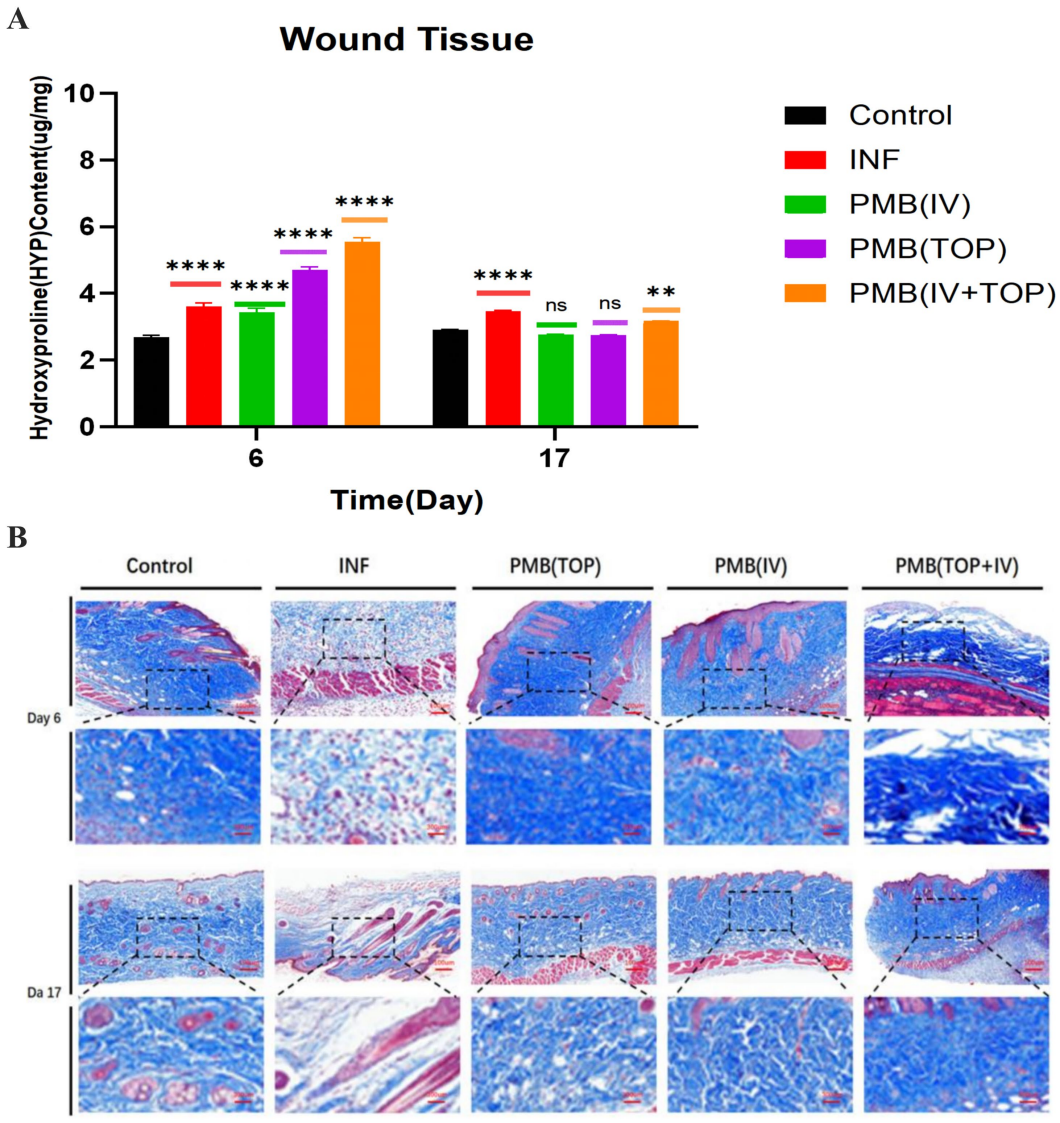
The effect of PMB administered via different routes on collagen deposition in mouse wound tissue. **(A)** Hydroxyproline (HYP) content in wound tissue from mice in each group on days 6 and 17 post-surgery. Data are presented as mean ± SD; *n* = 3 biological replicates (mice) per group per time point, each with three technical replicates (****p ≤ 0.0001, ***p ≤ 0.001, **p ≤ 0.01, *p ≤ 0.05, ns: *p* > 0.5). **(B)** Masson’s trichrome staining of mouse wound tissue on days 6 and 17 post-surgery.

The quantification of hydroxyproline (HYP), a well-established marker for collagen metabolism, was utilized to evaluate the effects of PMB therapy on extracellular matrix synthesis during the wound healing process (Figure 4A). At the early proliferative phase (day 6), HYP levels in all infected groups were elevated relative to the uninfected Control group (2 0.7 μg/ mg tissue). The INF group exhibited an increase (3 0.6 μg/ mg), indicating dysregulated initial matrix deposition amidst ongoing inflammation. The PMB (IV + TOP) group exhibited the highest HYP concentration (5 0.5 μg/ mg), indicating that it exerts a strong early stimulatory effect on fibroblast activity. The PMB (TOP) group also demonstrated a significant increase (4.7 μg/ mg), while the PMB (IV) group presented a more moderate rise (3.4 μg/ mg). By the remodeling phase (day 17), distinct patterns emerged. The INF group maintained a relatively high HYP level (3 0.5 μg/ mg), potentially reflecting disorganized and persistent collagen deposition. In contrast, the treated groups, particularly PMB (TOP) and PMB (IV), showed a marked reduction in HYP compared to their respective day-6 values (both decreasing to 2.8 μg/mg). This is consistent with active collagen maturation and remodeling. Although HYP levels in the PMB (IV + TOP) group also decreased by day 17, they remained the highest among treatment groups at 3.2 μg/ mg, indicating a distinct remodeling kinetic profile.

Masson’s trichrome staining was employed to assess collagen architecture, which further validated the biochemical observations at the histological level (Figure 4B). On day 6, wounds from the INF group exhibited sparse and disorganized collagen fibers (stained blue), primarily localized to wound margins amidst a background dominated by inflammatory cells (red). The PMB (TOP) and PMB (IV) groups displayed more abundant and organized collagen deposition within developing granulation tissue, forming an early reticular network. The PMB (IV + TOP) group showed moderate collagen deposition. By day 17, critical differences in tissue maturity were apparent. The INF group displayed fragmented, wavy collagen bundles with large, disordered gaps, consistent with immature scar tissue. In contrast, the PMB (TOP) and PMB (IV) groups exhibited the most mature collagen architecture, characterized by dense, thick, and parallel-aligned collagen bundles that resembled normal dermal structure. The PMB (IV + TOP) group also showed well-deposited collagen, though with slightly less uniform bundle alignment and density compared to the TOP and IV monotherapy groups.

## 4 PMB treatment modulates the expression of key wound healing cytokines

To elucidate the immunomodulatory mechanisms underlying the differential healing outcomes, we analyzed the expression dynamics of pivotal cytokines: the pro - inflammatory mediators interleukin-1*β* (IL-1β) and tumor necrosis factor-*α* (TNF-α), and the pro-fibrotic cytokine transforming growth factor-*β* (TGF-β).

### 4.1 Attenuation of pro-inflammatory cytokines IL-1β and TNF-α

**Figure 5:**
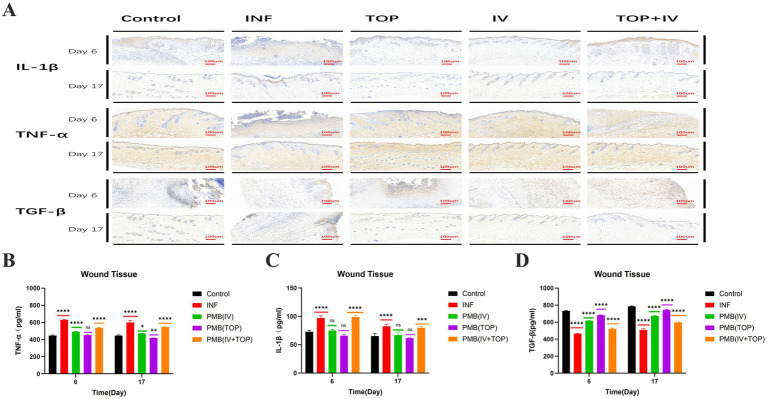
A study on the effects of polymyxin B treatment on inflammatory factors in wound tissue in mice. **(A)** Immunohistochemical staining for the cytokines IL-1*β*, TNF-*α* and TGF-β was performed on wound tissue on days 6 and 17 post- surgery. **(B)** Quantitative analysis of the cytokine TNF-α in wound tissue was performed on days 6 and 17 post-surgery. **(C)** Quantitative analysis of interleukin-1β (IL-1β) in wound tissue was performed on days 6 and 17 post-surgery. **(A)** Quantitative analysis of the cytokine TNF-α in wound tissue was performed on days 6 and 17 post- surgery. **(D)** Quantitative analysis of the wound tissue cytokine TGF-β was performed on days 6 and 17 post-surgery. Data are presented as mean ± SD; *n* = 3 biological replicates (mice) per group per time point, each with three technical replicates (****p ≤ 0.0001, ***p ≤ 0.001, **p ≤ 0.01, *p ≤ 0.05, ns: *p* > 0.5).

Persistent infection in the INF group sustained a heightened pro-inflammatory state. This was evidenced by significantly elevated tissue concentrations of IL-1 *β* and TNF- α at day 6, which remained abnormally high through day 17 (Figures 5B,C). Immunohistochemistry (IHC) for IL-1*β* and TNF-α (Figure 5A) confirmed this pattern, showing intense and widespread positive staining primarily localized to infiltrating leukocytes in INF group sections at both time points. All PMB treatment regimens effectively suppressed this excessive inflammation, albeit to varying degrees. At day 6, the PMB (TOP) and PMB (IV) groups exhibited the strongest anti-inflammatory effects. Compared to the INF group, they reduced IL-1*β* levels by approximately 31 and 22%, and TNF-α levels by 18 and 14%, respectively, compared to the INF group (*p* < 0.0001). I HC revealed a marked reduction in staining intensity and area for both cytokines in these groups. The PMB (IV + TOP) group showed a more modest reduction. By day 17, the PMB (TOP) group demonstrated the most complete resolution of inflammation, with IL-1*β* and TNF-α concentrations approaching those of the uninfected Control group (*p* < 0.0001 vs. INF). Corresponding I HC slides showed only sparse, faint positivity. In contrast, the INF group and, to a lesser extent, the PMB (IV + TOP) group, still displayed significant cytokine expression, indicating a failure to fully resolve the inflammatory phase.

### 4.2 Regulation of TGF-β expression

TGF-β, a master regulator of fibroblast activity and matrix synthesis, exhibited a distinct expression profile (Figure 5D). At the early phase (day 6), the INF group showed suppressed TGF-β levels (464 pg./mg) compared to the Control group (731.4 pg./mg). All PMB-treated groups counteracted this suppression. The PMB (TOP) and PMB (IV) groups elevated TGF-β to 682 and 616 pg./mg, respectively (*p* < 0.0001 vs. INF). IHC for TGF-β at day 6 showed strong positive staining in fibroblasts and macrophages within the granulation tissue of these treatment groups. By the later remodeling phase (day 17), a critical divergence was observed. While the INF group’s TGF-*β* level increased (507 pg./mg), it remained dysregulated. The PMB (TOP) group achieved an optimal TGF-β level (742 pg./ mg), significantly higher than the INF group and all other treatment groups (*p* < 0.0001). The PMB (IV + TOP) group showed an increase to 595 pg./ mg. The sustained yet appropriately localized elevation of TGF-β in the TOP group, as visualized by I HC, suggests a temporally and spatially regulated signaling profile conducive to quality tissue regeneration.

## 5 Discussion and conclusion

**Figure 6:**
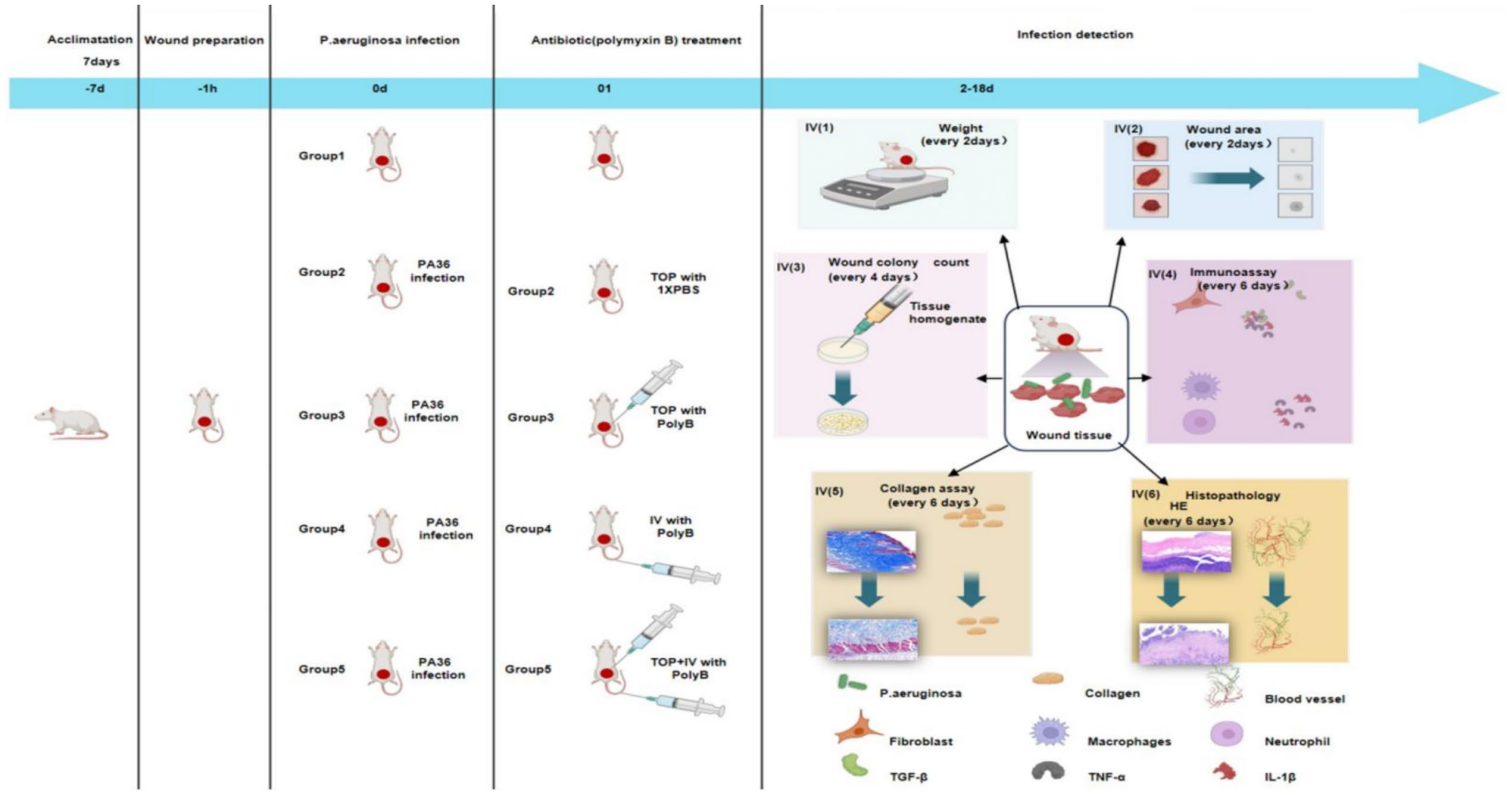
Process for establishing a mouse wound infection model with *Pseudomonas aeruginosa* treated with polymyxin B.

This study provides a comprehensive, multi-parameter evaluation of the efficacy of different PMB administration routes in a murine model of XDR-PA full - thickness wound infection (Figure 6). Contrary to the initial hypothesis that combined therapy might yield synergistic benefits, our data consistently demonstrate a clear hierarchy of therapeutic efficacy: topical monotherapy [PMB(TOP)] > intravenous monotherapy [PMB(IV)] > combined therapy [PMB(TOP+IV)], with the untreated infection (INF) group faring the worst. The superior performance of topical PMB is evident across all measured endpoints. It achieved the most rapid and complete wound closure, the most potent bactericidal effect (near sterilization by day 12), and the most favorable modulation of the wound microenvironment. This was characterized by an early and robust induction of reparative processes, evidenced by elevated total protein and hydroxyproline content at day 6. This was followed by an efficient transition to tissue remodeling, as seen in the maturation of collagen architecture and the resolution of pro- inflammatory cytokines (IL-1 *β*, TNF-*α*) by day 17. The ability of topical delivery to achieve and sustain extremely high local drug concentrations at the infection site, directly overcoming the biofilm barrier and the localized nature of the infection, is the most plausible explanation for its supremacy (Lin et al., 2018). Notably, topical administration minimizes systemic drug exposure—a key advantage given the inherent nephrotoxicity and neurotoxicity of PMB that limit its systemic use. By concentrating at the wound site with minimal systemic absorption, the drug exerts milder effects on systemic physiology.

Beyond these pharmacokinetic advantages, emerging evidence suggests that topical PMB exerts multi-level regulatory effects on the wound microenvironment, encompassing signal transduction, epigenetic modification, metabolic reprogramming, and cellular communication. In the inflammatory signaling network, IL-1 *β* and TNF- α primarily mediate tissue damage and delayed repair through activation of the NF- *κ* B pathway. Lipopolysaccharide (LPS) derived from Gram-negative bacteria activates TLR4 signaling, which is a key driver of excessive inflammation in infected wounds. Recent studies have demonstrated that PMB can bind LPS and block TLR4/MD-2 complex formation, thereby inhibiting downstream MyD88-dependent signaling (Huang et al., 2025). This mechanism may explain why topical PMB achieves superior anti-inflammatory effects compared to systemic administration, as higher local concentrations enable more complete TLR4 pathway suppression.

Regarding repair signaling, the temporal regulation of the TGF_β/Smad axis is critical. The topical PMB group exhibited a significant increase in TGF_β on day 6, initiating fibroblast activation and collagen synthesis, followed by a moderate decline by day 17, consistent with an ideal “early initiation, late resolution” pattern. Mechanistically, PMB may facilitate Smad7-dependent negative feedback regulation, preventing excessive TGF_β signaling that could otherwise lead to fibrosis (Liu et al., 2022). It should be emphasized that the aforementioned molecular mechanisms (such as inhibition of the TLR4 pathway and regulation of the TGF-β/Smad axis) are hypotheses proposed on the basis of the mechanisms of PMB action reported in the literature; this study did not directly detect these molecular events. Future research should employ methods such as Western blotting, immunoprecipitation or specific inhibitors to directly validate the role of these pathways in local PM B therapy.

Recent studies have shown that PMB-based formulations can modulate M1/M2 macrophage polarization—promoting pro-inflammatory M1 responses in the early healing phase while facilitating transition to anti-inflammatory M2 phenotypes in later stages—thereby orchestrating the dynamic inflammatory balance required for optimal wound repair (Cui et al., 2024). This study did not conduct the relevant tests; in future research, we hope to validate these findings using methods such as immunofluorescence colocalization. A critical and unexpected finding was the inferior outcome of the combined (IV + TOP) therapy group. Given that this group received half the dose via each route (summing to the same total daily dose as monotherapy groups), the results suggest that neither route achieved a pharmacodynamically effective concentration at the wound site under this split-dosing regimen. For concentration-dependent antibiotics like PMB, the ratio of the area under the concentration-time curve to the minimum inhibitory concentration (AUC/MIC) is a key determinant of efficacy (van der Meijden et al., 2023). Halving the topical dose likely resulted in sub-inhibitory concentrations in deeper tissue layers, while halving the intravenous dose reduced systemic drug exposure, compromising its ability to supplement the local effect. This underscores that the benefit of route combination is not inherent but is contingent upon each component dose being sufficient to reach its PK/PD target at the site of infection (Mahadevan et al., 2026). The worsened systemic health observed in the combined therapy group arose from a dual burden: subinhibitory local doses failed to clear the XDR-PA infection, leading to prolonged inflammatory stress, while the intravenous component introduced systemic PMB toxicity. Consequently, these animals experienced drug-induced metabolic strain without achieving infection control.

Critical limitation regarding the interpretation of combination therapy: In this study, the combination therapy group received a total daily dose of 5 mg/kg (2.5 mg/kg topical + 2.5 mg/kg intravenous), which was equal to the total daily dose administered to the PMB (TOP) and PMB (IV) monotherapy groups (5 mg/kg via a single route). Consequently, the combination group received a lower dose per route of administration than each monotherapy group. This equal-total-dose design was intentionally chosen to compare the efficacy of administration routes under the same total drug load and to model a potential clinical strategy aimed at reducing systemic exposure. However, it precludes any direct conclusion about whether combination therapy would be synergistic, additive, or antagonistic compared to full-dose monotherapy. Our observation that the combination group did not outperform the monotherapy groups should be interpreted solely within the context of this equal-dose design. Future studies employing a factorial design (e.g., full-dose monotherapy versus full-dose combination) are necessary to definitively assess the potential of combination therapy.

The intermediate efficacy of intravenous monotherapy highlights the limitation of systemic administration for localized, biofilm-associated infections. While it provided significant benefit over no treatment, its slower bacterial clearance and wound healing kinetics likely reflect lower effective drug concentrations penetrating the wound bed from the circulation. This is a known challenge in the pharmacokinetics of systemically administered polymyxins for soft tissue infections (Slingerland and Martin, 2024). Additionally, systemic intravenous administration of PMB carries a higher risk of nephrotoxicity and neurotoxicity due to increased drug exposure, which may partly explain the less favorable systemic physiological outcomes observed in the PMB(IV) group compared with the topical group.

This study used outbred Kunming mice, whereas the majority of published wound infection models employ inbred strains (C57BL/6 or BALB/c). While the genetic diversity of outbred mice may better simulate clinical heterogeneity, it also limits direct comparability with existing literature. Key findings should be validated in standard inbred strains in future studies.

A significant limitation of this study is the absence of direct renal function assessment (e.g., serum creatinine, blood urea nitrogen, renal histopathology). Given that nephrotoxicity is the major dose-limiting adverse effect of systemic polymyxin B (Eljaaly et al., 2021; Kelesidis and Falagas, 2015), the potential renal-sparing benefit of topical administration, a key rationale for this route, remains to be directly demonstrated. While our data show superior local efficacy with topical PMB, we cannot conclude that topical administration reduces systemic toxicity without direct renal function measurements. Future studies should incorporate serial renal function monitoring to evaluate the safety profile of different administration routes.

While the presence of biofilms was cited as a key rationale in the introduction, this study did not perform direct structural confirmation of biofilm formation (e.g., Congo red staining, crystal violet assay, confocal laser scanning microscopy). The Giemsa staining employed here visualizes bacterial distribution within tissue but cannot resolve the extracellular polymeric matrix characteristic of biofilms. The observed persistent bacterial burden, CFU rebound following an initial decline, and delayed wound healing in untreated infected mice provide indirect functional evidence consistent with biofilm-associated infection (Ciofu and Tolker-Nielsen, 2019). However, direct visualization of biofilm architecture is necessary to definitively establish this mechanism. Future studies should incorporate confocal microscopy and biofilm-specific staining to evaluate the antibiofilm efficacy of different PMB administration routes.

In conclusion, under the equal total dose design employed in this study, the efficacy of the com bi nation therapy group did not surpass that of the monotherapy group. However, as the dose for each route was halved in the combination group, this result cannot directly rule out the possibility of synergistic effects from combination therapy. Future studies will need to employ sufficient doses via both routes (e. g., 5 + 5 mg/kg) or dose regimens optimized based on pharmacokinetic / pharmacodynamic principles to scientifically evaluate the potential value of combination therapy. It maximizes target-site exposure while minimizing systemic drug load and associated toxicity risks. The failure of the half-dose combination regimen to outperform either monotherapy offers a crucial cautionary insight for clinical protocol design. Simply adding administration routes without ensuring each delivers an adequate dose may not confer advantage and could potentially compromise outcomes (Liang et al., 2023). These findings strongly advocate for the preferential consideration of optimized topical polymyxin B formulations in the management of non-disseminated, resistant Gram-negative wound infections. They also highlight the necessity of dose-optimization studies in any future development of combined -route therapies. However, animal models cannot yet fully reflect the pathophysiology of human disease. The therapeutic efficacy of PMB administered via different routes for treating wound infections in patients with multidrug-resistant *Pseudomonas aeruginosa* remains to be further validated. Future studies should evaluate combination therapy using pharmacokinetic-guided equal-intensity dosing regimens (e.g., doses based on equivalent local and systemic exposure).

## Data Availability

The datasets presented in this study can be found in online repositories. The names of the repository/repositories and accession number(s) can be found in the article/Supplementary material.
