# Supplementary material for: Comparative study on the wound healing effects of local versus systemic application of Polymyxin B in mice infected with multidrug-resistant Pseudomonas aeruginosa
Source: Front Microbiol. 2026 May 18;17:1812925. doi: 10.3389/fmicb.2026.1812925 (PMC13223157; doi:10.3389/fmicb.2026.1812925)
Supplement: Supplementary file 1 [file Data_Sheet_1.PDF]

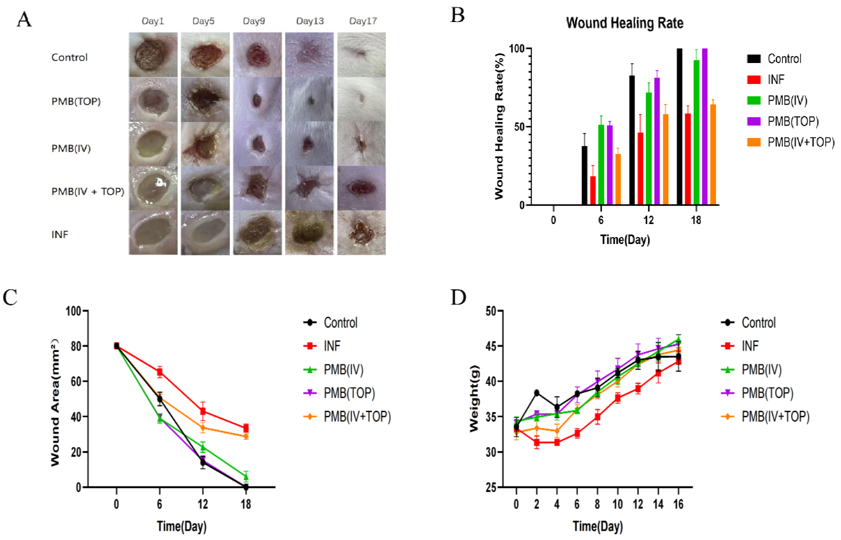

Figure 1： Differential Effects of Polymyxin B (PMB) Administration Routes on Wound Healing and Systemic Health in XDR-PA Infected Mice.A:Representative photographs of the wounds in each group of mice, taken on days 1, 5, 9, 13 and 17 following infection.B:Wound healing rates in each.C:Wound healing area in each group of mice following surgery.D:Weight changes in each group of mice every two days following surgery.Data are presented as mean ± SD; n = 3 biological replicates (mice) per group per time point, each with three technical replicates

.

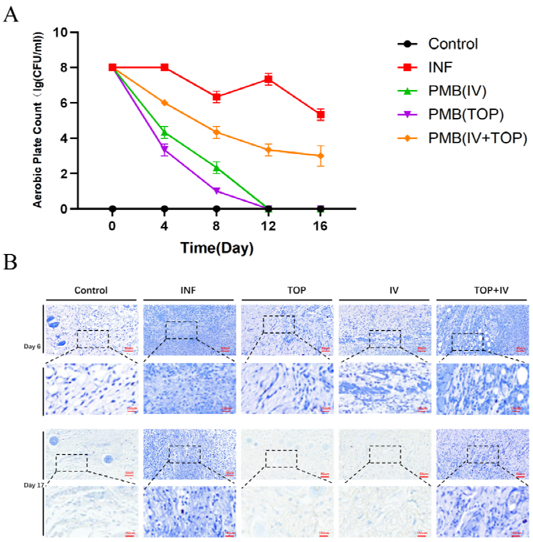

Figure 2:The effect of different routes of administration on bacterial load in wounds.A:Colony counts of *Pseudomonas aeruginosa* (PA36) in the postoperative wounds of mice in each group.B: Giemsa staining of wound tissue in mice on days 6 and 17 post-surgery.

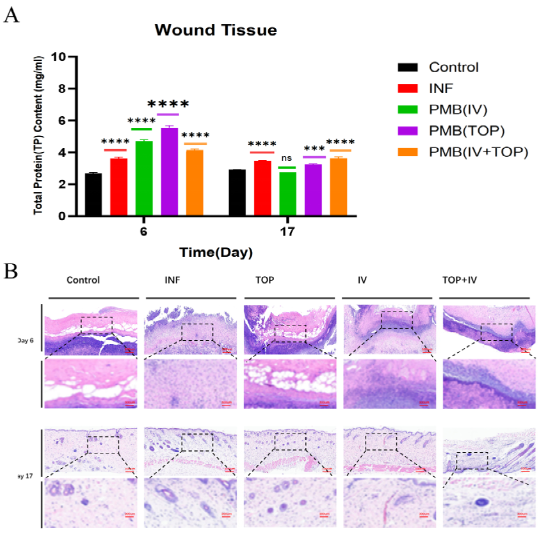

Figure 3： The effect of PMB on total protein content in wound tissue and granulation tissue formation.A：Total protein (TP) content in wound tissue from mice in each group on days 6 and 17 post-surgery. Data are presented as mean ± SD; n = 3 biological replicates (mice) per group per time point, each with three technical replicates（****： $p \leq 0.0001$,***： $p \leq 0.001$， **： $p \leq 0.01$， *： $p \leq 0.05$， ns： $p > 0.5$）.B:Hematoxylin and eosin staining was performed on wound tissue from the mice on days 6 and 17 post-surgery.

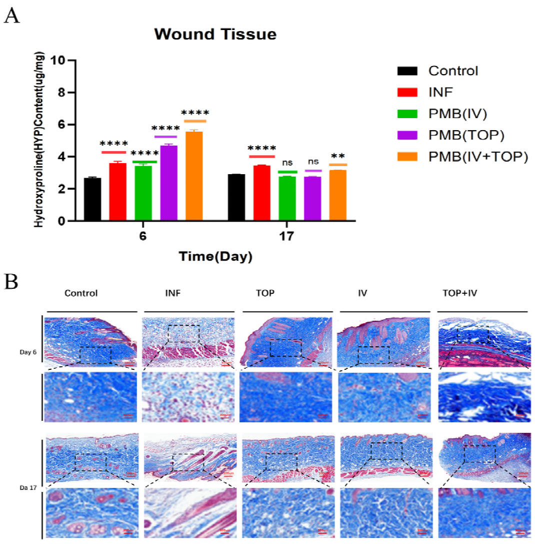

Figure 4： The effect of PMB administered via different routes on collagen deposition in mouse wound tissue.A：Hydroxyproline (HYP) content in wound tissue from mice in each group on days 6 and 17 post-surgery.Data are presented as mean ± SD; n = 3 biological replicates (mice) per group per time point, each with three technical replicates（****： $p \leq 0.0001$,***： $p \leq 0.001$， **： $p \leq 0.01$， *： $p \leq 0.05$， ns： $p > 0.5$）.B： Masson’s trichrome staining of mouse wound tissue on days 6 and 17 post-surgery.

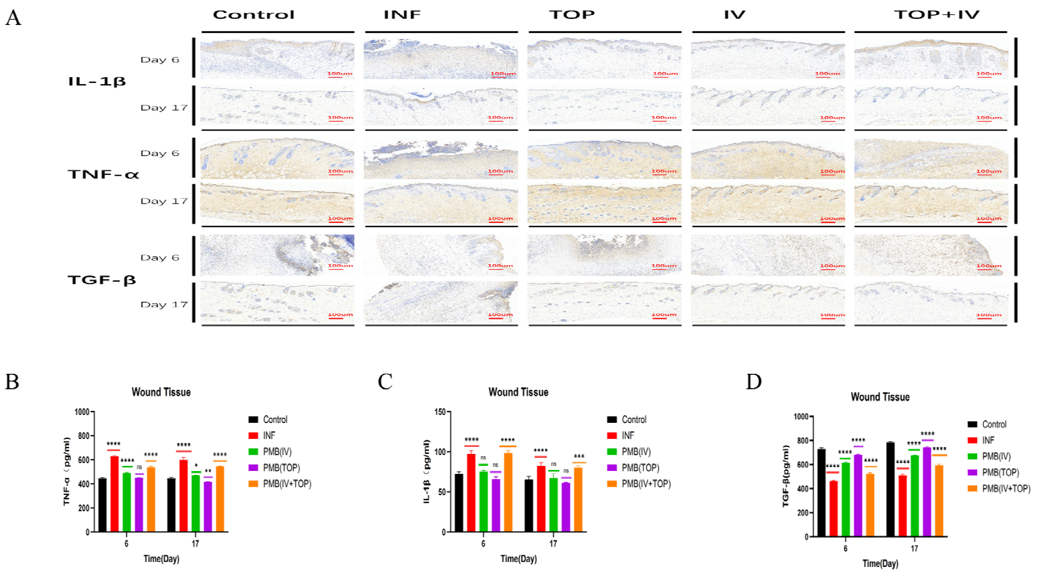

Figure 5： A study on the effects of polymyxin B treatment on inflammatory factors in wound tissue in mice.A: Immunohistochemical staining for the cytokines IL-1β, TNF-α and TGF-β was performed on wound tissue on days 6 and 17 post-surgery.B:Quantitative analysis of the cytokine TNF-α in wound tissue was performed on days 6 and 17 post-surgery.C: Quantitative analysis of interleukin-1β (IL-1β) in wound tissue was performed on days 6 and 17 post-surgery. A:Quantitative analysis of the cytokine TNF-α in wound tissue was performed on days 6 and 17 post-surgery.D:Quantitative analysis of the wound tissue cytokine TGF-β was performed on days 6 and 17 post-surgery.Data are presented as mean ± SD; n = 3 biological replicates (mice) per group per time point, each with three technical replicates（****： $p \leq 0.0001$,***： $p \leq 0.001$， **： $p \leq 0.01$， *： $p \leq 0.05$， ns： $p > 0.5$）.

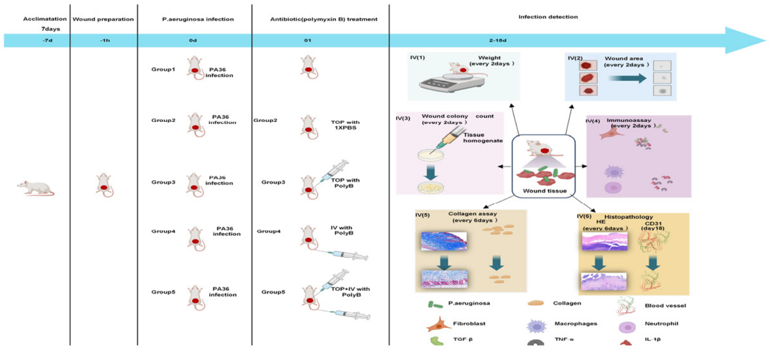

Figure 6：Process for establishing a mouse wound infection model with *Pseudomonas aeruginosa* treated with polymyxin B.

.
